# Changes in Muscle Quality and Gut Microbiota of Whiteleg Shrimp (*Penaeus vannamei*) Within a Live Supply Chain

**DOI:** 10.3390/ani15101431

**Published:** 2025-05-15

**Authors:** Ping Zhang, Zian Jiang, Yuwei Zhang, Lele Leng, Ziyi Yin, Weining He, Xiaoqun Zeng, Daodong Pan

**Affiliations:** 1 State Key Laboratory for Quality and Safety of Agro-Products, Ningbo University, Ningbo 315211, China; 2 College of Food Science and Engineering, Ningbo University, Ningbo 315800, China; 3 Zhejiang-Malaysia Joint Research Laboratory for Agricultural Product Processing and Nutrition, Ningbo University, Ningbo 315800, China

**Keywords:** *Penaeus vannamei*, live supply chain, muscle quality, gut microbiota, stress response, low temperature, biomarker

## Abstract

During farm-to-consumer transport, a live supply chain can aid in maintaining the quality of whiteleg shrimp (*Penaeus vannamei*). However, the changes in muscle quality and gut microbiota of shrimp in the live supply chain and their interactions are poorly understood. Here, we investigated the dynamics of cumulative survival, muscle quality, and gut microbiota in the key phases of the live shrimp supply chain: post-harvest, post-transport, post-respite, and simulated sales [ambient temperature (AT; 29 °C ± 0.3 °C); low temperature (LT; 23 °C ± 0.3 °C)]. The results suggest that among the various stages, the highest mortality (12%) occurred after transport, while the respite process was associated with enhanced gut-mediated stress resilience. Notably, the transport, 24 h sales, and 40 h sales stages were identified as three potential critical control points. Furthermore, the LT group exhibited an 8% higher survival rate, better quality parameters (34.9% higher hardness), increased abundance of Bacteroidetes (from 3.63% to 7.39%), and a reduced F: B ratio. Correlation analysis identified *Xanthomonadales* and *Oscillospirales* as potential biomarkers for maintaining quality, positively linked to survival, muscle hardness, and brightness. Our findings provide valuable insights into optimizing control strategies and microbial biomarkers for enhancing muscle quality in live supply chains and aquaculture.

## Simple Summary

This study tracked the critical phases of the live shrimp supply chain, including post-harvest, post-transport, post-respite, and simulated sales under two temperature conditions: ambient temperature (AT; 29 °C ± 0.3 °C) and low temperature (LT; 23 °C ± 0.3 °C). The aim was to address the gap in changes in the muscle quality and gut microbiota of shrimp throughout the live supply chain and their interactions. The results suggest that transport was associated with the highest mortality and altered metabolic priorities, while shrimp subjected to a 3 h respite showed signs of stress relief and beneficial shifts in gut microbiota function. The LT group exhibited higher survival rates, better quality parameters, and more stable gut microbiota. Furthermore, *Xanthomonadales* and *Oscillospirales* were identified as potential biomarkers for maintaining shrimp quality. These findings offer insights into optimizing shrimp supply chain strategies and propose gut microbiota biomarkers for improving muscle quality in aquaculture management.

## 1. Introduction

The whiteleg shrimp (*Penaeus vannamei*) is one of the most commercially valuable crustaceans cultivated globally [1,2]. In 2022, global shrimp aquaculture production reached 6.83 million t, a 7.7% increase from 6.34 million t in 2021 [3]. Currently, shrimp are distributed through two main supply chains: live and frozen storage. Numerous studies have documented that frozen storage adversely affects shrimp quality, including myofibrillar deformation [4], loss of organoleptic quality [5], increased thiobarbituric acid (TBA) reactive substance (TBARS) content [6], and the formation of black spots after thawing [7]. Although more expensive than frozen shrimp, live shrimp are favored by consumers for their superior sensory quality and nutritional retention compared with frozen alternatives [8], driving a steady rise in demand. Thus, in “farm-to-fork” live supply chains (including harvesting, transport, respite, and sale), maximizing shrimp survival and maintaining quality are critical.

When shrimp move through a live supply chain, they encounter a variety of unavoidable stress conditions that are likely to combine to trigger physiological deterioration, loss of muscle quality, and microflora dysbiosis [9]. Previous studies have highlighted factors influencing post-harvest quality loss in shrimp and fish, such as prolonged transport [10], overcrowding [11], oxygen fluctuation [12], respite periods before shipment [13], and temperature variations during transport [14]. Meanwhile, the gut microecosystem serves as a key regulator of host activity, and the proposed “gut–muscle axis” theory provides a fundamental basis for understanding the interactive effects between the gut microbiota and muscle quality [15]. Studies have reported that dietary interventions can improve muscle quality by regulating gut microbiota-mediated nutrient metabolism and immune responses [16,17]. Moreover, the gut microecosystem undergoes stress-induced changes that impair nutrient absorption and antioxidant functions, leading to muscle deterioration, primarily through accelerated lactic acid accumulation (from glycolysis) and lipid oxidation [18,19]. Recent studies on shrimp gut microbiota have focused on tracking various stressful situations in the culture process, such as salinity changes [20], nutritional composition [21], growth temperature [22], and probiotic feeding [23]. Overall, most existing studies focus on isolated stages of the supply chain or single stress factors, with significant gaps in understanding how different stages of the live supply chain affect muscle quality, microbiota, and their interactions, particularly in *P. vannamei*.

To address this gap, we tracked and simulated various phases of the practical live supply chain for whiteleg shrimp distribution, which includes post-harvest (PH), post-transport (PT), post-respite (PR), and simulated sales phases [ambient temperature (AT), 29 °C ± 0.3 °C, and low temperature (LT), 23 °C ± 0.3 °C]. The cumulative survival rate, muscle quality parameters [i.e., color, texture, pH, lactate content, and TBARSs], and gut microbiota [i.e., Chao1 and Simpson indexes, principal component analysis (PCA) plot, community structure, and function prediction] were assessed. By integrating and analyzing these datasets, we aimed to investigate changes in the quality and intestinal bacterial communities of *P. vannamei* throughout the live supply chain, focusing particularly on the effects of temperature fluctuations. Based on the analysis of muscle quality and gut microbiota changes in *P. vannamei* in the live supply chain, this study provides critical control points and key microbial biomarkers associated with high muscle quality, aiming to improve the survival rates and extend the shelf life of live *P. vannamei* in post-harvest and aquaculture management.

## 2. Materials and Methods

### 2.1. Experimental Design and Supply Chain Procedure

Shrimp were reared in ponds under the following conditions: 25–33 °C, 17–19 ppt salinity, pH 7.6–8.3, and ≥5.2 mg/L dissolved oxygen. They were fed primarily (>70%) commercial feed (Ningbo Zhengda Agriculture Co., Ltd. (Ningbo, China)), supplemented with natural prey (algae) and animal-based nutrients. After about 140 days of rearing at Ningbo Zhengda Agriculture Co., Ltd. (Ningbo, China), shrimp were harvested by using a dragnet from a single pond and production batch. After discarding weak or low-activity individuals, a total of 1220 healthy shrimp (14.2 ± 2 g) were selected for transportation trials. Three squared plastic containers (equal volumes of pond water with equivalent shrimp biomass) were utilized for transport. Throughout the 30 min transit to Ningbo University’s Meishan Campus, conditions were maintained at a temperature of 26.5–28 °C, salinity of 17–19 ppt, dissolved oxygen of 3.9–4.8 mg/L, and biomass density of 385–400 g/L, monitored continuously by using a WTW Multi 340i/SET multiparameter system (JIAWU Automation Technology Co., Ltd., Shanghai, China).

The respite and simulated sales process were carried out in a temperature-controlled indoor aquaculture system (Guangzhou HuanKong Agricultural Biotechnology Co., Ltd., Guangzhou, China) at the pilot facility. The system consisted of 12 independent plastic drums (capacity = 600 L, diameter = 80 cm, and height = 120 cm) containing seawater without substrate, with water circulation being maintained through biological filtration. Each treatment group consisted of three replicate plastic drums. During the respite and simulated sales phases, environmental conditions were maintained as follows: salinity of 24.0–25.3 ppt, pH 7.6–7.9, dissolved oxygen of 5.2–5.6 mg/L, and biomass density of 90–100 g/L. After a 3 h respite at 29 °C, the shrimp were randomly divided into two temperature groups for a 48 h simulated sales experiment: AT and LT. The “Shenzhen Quality Food—Code of Good Practice for Fresh Aquatic Products” (T/SZS 3016-2020) [24] recommends a temperature between 20 and 22 °C during the fasting respite of shrimp. Based on this guideline and with minor adjustments, the simulated sales phase in this study set the AT (29 °C ± 0.3 °C) group as the untreated water temperature group and the LT (23 °C ± 0.3 °C) group as the cooled temperature group.

**Figure 1:**
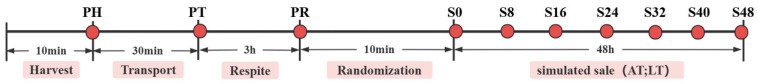
Schematic diagram of the entire post-harvest supply chain for live shrimps. Harvested shrimps are transported, given a respite, randomly assigned, and then allowed to enter to the sales phase. Red dots indicate sampling time points. PH: post-harvest; PT: post-transport; PR: post-respite; S0, S8, S16, S24, S32, S40, and S48: different time points in the simulation of the sales stage, which are 0, 8, 16, 24, 32, 40, and 48 h respectively; AT: ambient temperature; LT: low temperature.

Following the T/SZS 3016-2020 guidelines for live aquatic product cold chains, the entire live shrimp supply chain is schematized in Figure 1.

### 2.2. Sample Collection

For each sampling event, a total of 30 live shrimp were randomly selected from the three containers, with equal representation from each container (10 shrimp per container). All shrimp were rapidly euthanized through ice burial, a process that takes about 1–2 min, followed by soaking in 75% ethanol for 2 s and rinsing immediately. Gut and tail muscle samples were collected separately after shelling and head removal.

Muscle sampling was performed at PH, PT, and PR, as well as at 0, 8, 16, 24, 32, 40, and 48 h during the simulated sales period (denoted by S0, S8, S16, S24, S32, S40, and S48, respectively). To minimize postmortem changes, color and texture measurements were completed within 30 min of ice-immersion euthanasia. Moreover, intestinal samples were collected at PH, PT, and PR, as well as from the AT and LT group shrimp at S0, S24, and S48. To minimize the effects of individual variation, intestinal samples from 10 shrimp were pooled as one biological replicate, with three such replicates being analyzed per experimental group. The intestinal and meat samples were obtained in a sterile environment, rapidly frozen in liquid nitrogen, and stored at −80 °C for subsequent quality assessments (TBARSs, pH, and lactate) and 16S rRNA analysis.

### 2.3. Cumulative Survival Rate Analysis

Following previously reported methods [25] with minor modifications, we assessed shrimp behavior and appearance to determine viability based on the following factors: (1) lying on the side at the bottom and unresponsive to water flow and external stress; (2) dried, damaged, or missing eyes; (3) damage to or absence of head carapace and abdominal legs; and (4) completely gray tail muscles and completely opaque appearance. Dead shrimp were removed, weighed, and counted. The cumulative shrimp survival rate was calculated as follows:

(1) $$\mathrm{Cumulative}\;\mathrm{survival}\;\mathrm{rate}\;(\%)=\left[1-(\frac{\sum N_{1}}{N_{0}})\right]\;\times \;100\%,$$

where N_0_ refers to the number of shrimp at PH and N_1_ is the number of dead shrimp observed during the experiment.

### 2.4. Muscle Quality Analysis

#### 2.4.1. Color Characteristic Analysis

Color measurements were performed on a portable colorimeter (CR-400; Konica Minolta, Tokyo, Japan). The first muscle sections were cut with scissors to record the values of L* (brightness), a* (reddish-green color), and b* (yellowish-blue color). The instrument was calibrated by using black and white standard plates (L* = 55.02, a* = −1.32, b* = 14.83). Six samples were measured thrice per treatment, with freshly caught shrimp (PH) used as the reference to calculate the total color difference (∆E*) as follows [26]:

(2) $$∆E^{*}\;=\sqrt{∆L^{*2}+∆a^{*2}+∆b^{*2}},$$

#### 2.4.2. Texture Profile Analysis of Fresh Muscle

Texture profile analysis (TPA) was conducted by using a TA.XT Plus Texture Analyzer (Stable Micro Systems, Surrey, UK) following a previously established method [27], with slight modifications. The second abdominal muscle was cut into a square by using disinfected scissors and placed on the plate of the texture analyzer with a flat-bottomed cylindrical probe (P/50 Characterization). For each group, six samples were tested with two repeated compression measurements per sample. The test conditions were as follows: pre-test speed, 2 mm/s; mid-test speed, 1 mm/s; post-test speed, 1 mm/s; dwell interval, 5 s; point of impact force, 5 g; and compression degree, 30%.

The following texture parameters were derived: hardness (N), defined as the maximum force during the first compression; gumminess (N), calculated as hardness multiplied by a constant derived from instrument software based on the shape of the force–time curve; chewiness (mJ), calculated as gumminess multiplied by springiness; and springiness (mm), the distance the sample recovered after the first compression.

#### 2.4.3. TBARS Content in Muscle

Muscle TBARS content, expressed as milligrams of malondialdehyde (MDA) per kilogram of the sample, was determined based on the spectrophotometric method of China’s National Food Safety Standard “Determination of Malondialdehyde in Foods” (GB 5009.181-2016) [28], with slight modifications. Mixed extracts (*w*/*v*) at a concentration of 75% trichloroacetic acid (TCA) and 1.0% ethylene diamine tetraacetic acid (EDTA) were configured, mixed, and stored at room temperature. A 20 mL aliquot of mixed extraction solution was added to 2 g of pooled shrimp muscle samples. Homogenization was performed for 30 s at 8500 rpm by using an FSH-2A homogenizer (Xunsheng Instrument Co., Ltd., Changzhou, China) with ice-bath cooling. Next, we sealed the mixture with a plug and placed it on an HWM-3S8 thermostatic shaker (Jiangnan Instruments, Ningbo, China) at 50 °C for 30 min. The extract (5 mL) was filtered through a double-layered quantitative slow filter paper, mixed with an equal amount of 0.02 M TBA, and reacted in a water bath at 90 °C for 30 min. The absorbance of the cooled solution was measured at 532 nm on an Infinite M200PRO Multifunctional Enzyme Spectrometer (TECAN, Salzburg, Austria). Finally, the TBARS content was calculated by using a standard curve.

#### 2.4.4. pH and Lactate Content

pH was determined by using a method described in the literature [29], with slight modifications. Briefly, 2 g of the sample was homogenized with 20 mL of distilled water at 8000 rpm for 30 s by using an FSH-2A homogenizer (Xunsheng Instrument Co., Ltd., Changzhou, China) with ice-bath cooling. The homogenate was left standing for 30 min and then filtered. The pH of the filtrate was determined. Three biological replicates were tested per group, with pH measurements being performed in triplicate and averaged for each sample.

Lactate content was determined by using an L-Lactate Content Assay Kit (Solarbio, Beijing, China).

### 2.5. Gut Microbiota

In brief, the total genomic DNA of the intestinal samples (n = 3 per group) was extracted by using the SDS method. DNA integrity and concentration were then verified by using 1.5% agarose gel electrophoresis. The extracted DNA was then diluted to 1 ng/μL with sterile water and stored at −80 °C for subsequent use as a template. DNA was amplified through polymerase chain reaction (PCR) by using Phusion High-Fidelity PCR Master Mix with GC Buffer (New England Biolabs, Beijing, China). The reaction mixture contained 25 μL of 50 ng genomic DNA and the primers 515F and 806R (2.5 μL of each) [30]. The PCR conditions were adapted from previous studies [31] with modifications as follows: initial denaturation at 98 °C for 30 s, followed by 35 cycles of 98 °C for 10 s, 54 °C for 30 s, and 72 °C for 45 s and a final extension at 72 °C for 10 min. All PCR products were purified and analyzed through 2% agarose gel electrophoresis, and amplicons were recovered by using a GeneJET Gel Recovery Kit (Thermo Fisher Scientific, Waltham, MA, USA) [32].

The purified amplicons were sent to Beijing Novozymes Bioinformatics (Beijing, China) for library construction and sequencing. Library construction was carried out by using an NEB Next Ultra DNA Library Prep Kit for Illumina (New England Biolabs, Beijing, China). The library was quantified by using Qubit, and sequencing was performed on the Illumina MiSeq platform. To assess sequencing depth, rarefaction curves were generated and showed clear plateaus, indicating that the sequencing depth was sufficient to capture the microbial diversity within the samples. After stringent quality filtering, the valid sequences were clustered into Operational Taxonomic Units (OTUs) at a 97% similarity threshold [33]. Downstream analyses, including differential abundance analysis, α diversity (Chao1 and Simpson), β diversity, and PICRUSt2-based functional predictions, were executed in QIIME2 (version 1.9.1) and R (version 4.0.3). Linear discriminant analysis (LDA) effect size (LEfSe) analyses were conducted with thresholds of LDA values > 2.5 and *p*-values < 0.05.

### 2.6. Data Evaluation and Analysis

All experimental data were analyzed by using SPSS 19.0 (IBM Corp., Armonk, NY, USA). The homogeneity of variances was first verified through Levene’s test. For data meeting homogeneity assumptions (*p* > 0.05), one-way ANOVA followed by Duncan’s multiple range test was applied. Non-parametric Kruskal–Wallis ANOVA was employed for heteroscedastic data (*p* < 0.05). Correlation analysis was performed by using Spearman’s correlation. All the data values are expressed as means ± standard deviation (SD), with *p* < 0.05 indicating significant differences.

## 3. Results

### 3.1. Cumulative Survival Rates

**Figure 2:**
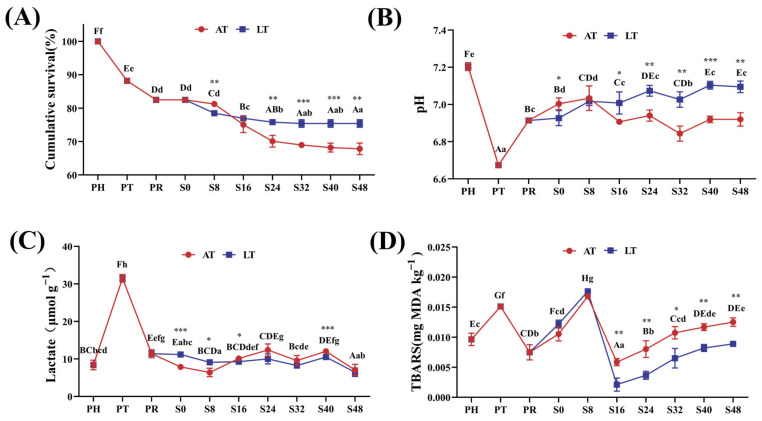
Cumulative survival rate of shrimps (**A**), and pH (**B**), lactate content (**C**), and TBARSs (**D**) in muscle. PH: post-harvest; PT: post-transport; PR: post-respite; S0, S8, S16, S24, S32, S40, S48: different time points in the simulation of the sales stage, which are 0, 8, 16, 24, 32, 40, and 48 h, respectively; AT: ambient temperature; LT: low temperature. Within-group differences are indicated by letters: upper case for LT and lower case for AT. Between-group differences are denoted by asterisks: * for *p* < 0.05, ** for *p* < 0.01, and *** for *p* < 0.001.

Figure 2A presents the changes in cumulative survival rates throughout the simulated supply chain. As the supply chain progressed, the cumulative survival rates of the shrimp gradually declined (*p* < 0.05). At PT and PR, the survival rates were 88.19% ± 0.33% and 82.48% ± 0.33%, respectively. During the simulated sales phase, both the AT and LT groups exhibited similar trends: From S0 to S24, shrimp mortality increased gradually, and after S24, the survival rates stabilized. Between S0 and S48, the AT group demonstrated a significant decrease in the survival rate from 81.26% ± 0.20% to 67.81% ± 1.75%; similarly, the LT group’s survival rate decreased from 82.48% ± 0.33% to 75.43% ± 1.43% during the same period. At S8, the survival rate was significantly higher in the AT group than in the LT group, but over S24–S48, the LT group’s survival rates remained consistently and significantly higher than those of the AT group (*p* < 0.01).

### 3.2. Changes in Muscle Quality

#### 3.2.1. pH and Lactate Content

Figure 2B shows changes in shrimp muscle pH over time. The pH significantly decreased from 7.19 ± 0.02 at PH to 6.67 ± 0.02 at PT (*p* < 0.001). At PR, the pH increased to 6.92 ± 0.02 but did not return to PH levels. During the simulated sales phase, both the AT and LT groups followed a similar pH trend: The pH increased from S0 to S8, decreased from S8 to S32, and increased again from S32 to S48. From S0 to S8, the pH was higher in the AT group, and over S0–S48, the AT group showed a 1.4% decrease, while the LT group showed a 2.5% increase (*p* < 0.05).

Figure 2C shows changes in muscle lactate content over time. Lactate content increased from 8.41 ± 1.25 μmol/g at PH to 31.48 ± 1.02 μmol/g at PT, followed by a sharp decrease at PR. From PR to S48, lactate levels remained relatively stable, fluctuating around 10 μmol/g in both AT and LT groups.

#### 3.2.2. Muscle TBARS Content

Figure 2D illustrates changes in TBARS content throughout the live supply chain. Overall, the TBARS levels remained relatively low during the entire process. At PH, muscle TBARS content was 0.0096 mg MDA/kg, which increased significantly to 0.0151 mg MDA/kg at PT and decreased significantly to 0.0074 mg MDA/kg at PR (*p* < 0.05). During the simulated sales phase, TBARS increased from S0 to S8, decreased from S8 to S16, and increased again from S16 to S48. Over S0–S48, TBARS content increased in the AT group, whereas it decreased in the LT group.

#### 3.2.3. Color Characteristic Analysis

**Figure 3:**
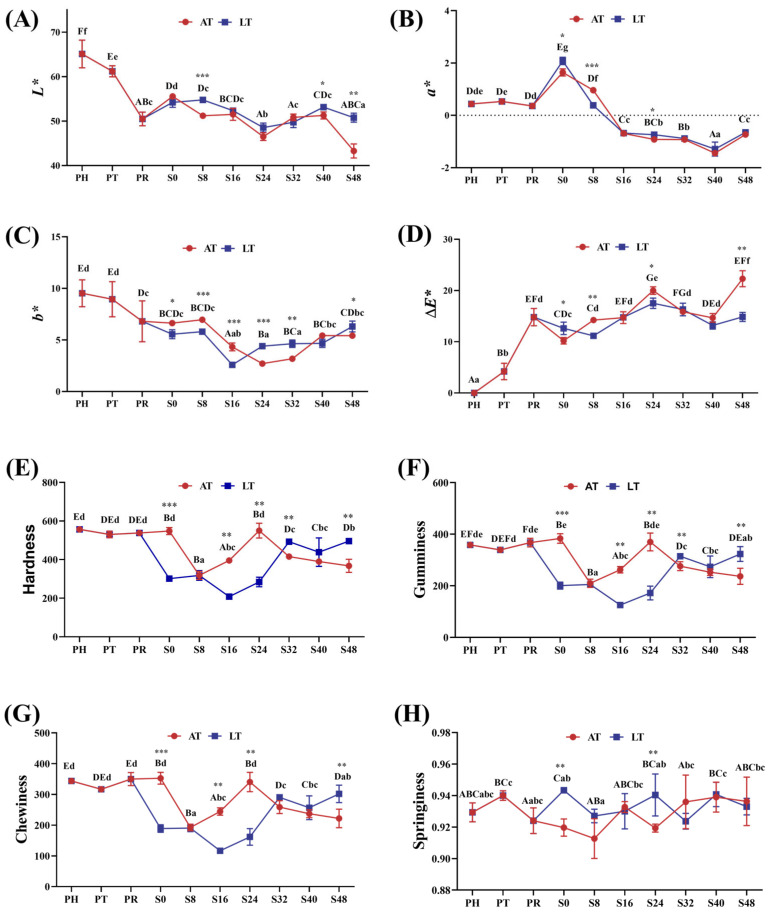
L* (luminance) value (**A**), a* (red-green) value (**B**), b* (yellow-blue) value, (**C**), ΔE* (total color difference) value (**D**), hardness (**E**), gumminess (**F**), chewiness (**G**), and springiness (**H**) in shrimp muscle. PH: post-harvest; PT: post-transport; PR: post-respite; S0, S8, S16, S24, S32, S40, S48: different time points in the simulation of the sales stage, which are 0, 8, 16, 24, 32, 40, and 48 h, respectively; AT: ambient temperature; LT: low temperature. Within-group differences are indicated by letters: upper case for LT and lower case for AT. Between-group differences are denoted by asterisks: * for *p* < 0.05, ** for *p* < 0.01, and *** for *p* < 0.001.

Figure 3A–D illustrate changes in shrimp muscle color parameters, which also reflect transparency variations.

At PT and PR, muscle transparency progressively decreased, as indicated by declining L* and b* values, no significant change in a*, and an increase in ∆E*. During the simulated sales phase, L* and b* exhibited a consistent trend: decreasing from S0 to S24, followed by an increase from S24 to S40. At S24 and S48, L* and b* in the LT group were significantly higher than those in the AT group, while ∆E* was lower (*p* < 0.05). After the 40 h mark, L* decreased, while a* and ∆E* increased. The LT group exhibited higher L* and b* but lower ∆E*, indicating better color stability and transparency.

#### 3.2.4. TPA

Figure 3E–H present the variations in hardness, gumminess, chewiness, and springiness throughout the live supply chain.

At PT and PR, there were no significant changes in hardness, gumminess, chewiness, and springiness. During the simulated sales phase, hardness, gumminess, and chewiness declined in the AT group but increased in the LT group—with the LT group demonstrating significantly higher values than the AT group at S48. However, springiness remained largely unaffected throughout this phase.

### 3.3. Changes in Gut Bacterial Communities of Shrimp

#### 3.3.1. Diversity Analysis

**Figure 4:**
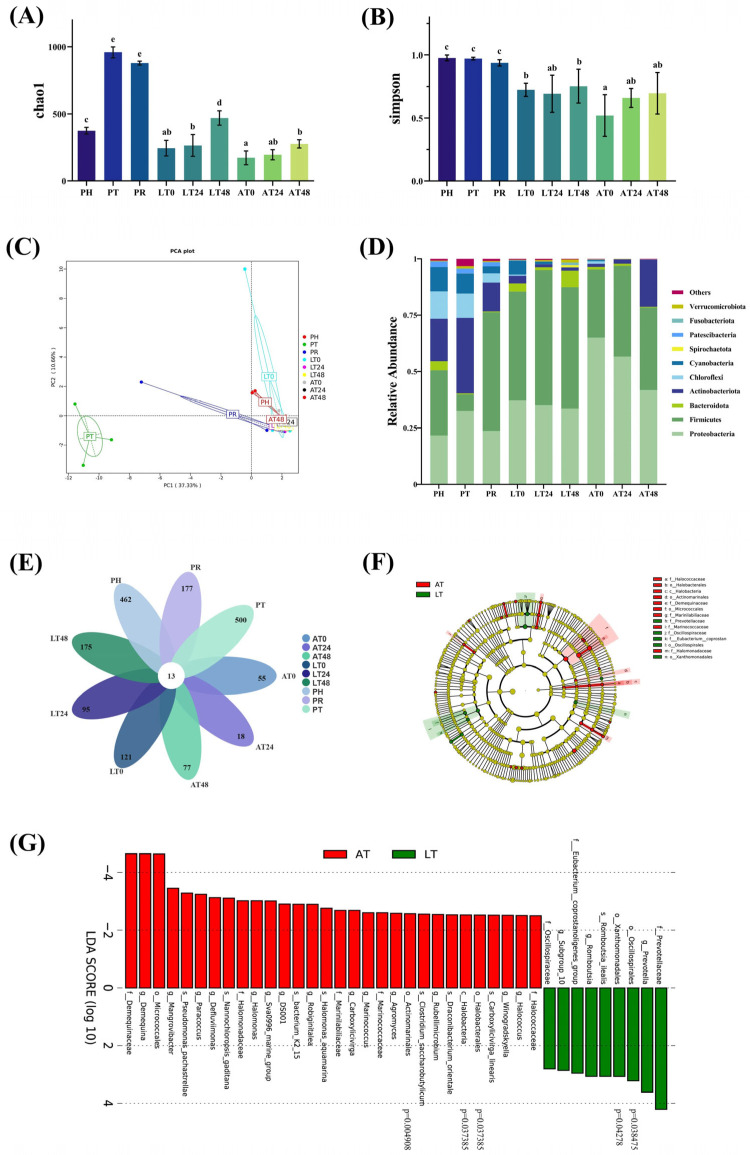
Diversity, species composition, and differential analysis of the intestinal bacterial community. Alpha diversity of gut microbiota evaluated by Chao 1 (**A**) and Simpson (**B**). PCA (**C**) was performed to compare gut microbial communities among all groups. Relative abundance of top 10 bacterial phyla (**D**) were determined based on 16S rRNA sequencing. Venn analysis of OTUs. (**E**). Evolutionary branching plot (**F**) and LDA score plot (**G**) for LEfSe analysis. PH: post-harvest; PT: post-transport; PR: post-respite; LT0, LT24, and LT48: 0, 24, and 48 h, respectively, in LT group; AT0, AT24, and AT48: 0, 24, and 48 h, respectively, in AT group. Different letters represent significant differences between groups (*p* < 0.05).

The community diversity (Simpson) and richness (Chao1) indices of shrimp intestines were analyzed. As shown in Figure 4A,B, the Chao1 index significantly increased at PT but decreased at PR, and no significant change in the Simpson index. They increased in both the LT and AT groups during the simulated sales phase. However, the Chao1 index was significantly higher at S48 in the LT group than in the AT group (*p* < 0.05), but the Simpson index did not differ significantly between the LT and AT groups over 0–48 h (*p* > 0.05). In general, at any time point, the Simpson index remained higher in the LT group than in the AT group.

Our PCA revealed that principal components PC1 and PC2 accounted for 37.33% and 10.66% of the variance, respectively, distinctly separating PT from other time points; the data at these other time points exhibited high clustering and intergroup similarity (Figure 4C).

#### 3.3.2. Community Structure and Differential Analysis

As illustrated in Figure 4D, overall, the top 10 relatively abundant phyla in the shrimp intestines were Proteobacteria, Firmicutes, Actinobacteria, Bacteroidetes, Chloroflexota, Cyanobacteria, Spirochaetota, Patescibacteria, Fusobacteriota, and Verrucomicrobiota. In freshly harvested shrimp (PH), we noted high relative abundance of Firmicutes (28.90%), Proteobacteria (21.63%), Actinobacteria (18.81%), Chloroflexota (12.16%), Cyanobacteria (10.77%), and Bacteroidetes (4.08%). At PT, the abundance of Proteobacteria (32.53%) and Actinobacteria (33.44%) increased, but that of Firmicutes (7.43%), Cyanobacteria (8.96%), Chloroflexota (10.80%), and Bacteroidetes (0.41%) decreased. At PR, the abundance of Proteobacteria (23.68%), Actinobacteria (12.79%), Chloroflexota (4.13%), and Cyanobacteria (3.06%) decreased, whereas that of Bacteroidetes increased slightly, and that of Firmicutes (52.61%) increased considerably. During the simulated sales phase, Firmicutes and Proteobacteria gradually became the dominant phylum. Over S0–S48, Proteobacteria abundance decreased from 65.07% to 41.85%, Firmicutes abundance increased from 30.31% to 36.59%, Bacteroidetes abundance decreased from 1.01% to 0.32%, and Actinobacteria abundance increased from 1.4% to 20.90% in the AT group. In contrast, in the LT group, Proteobacteria abundance decreased from 37.21% to 33.61%, Firmicutes abundance increased from 48.52% to 53.84%, Bacteroidetes abundance increased from 3.63% to 7.39%, and Actinobacteria abundance decreased from 3.41% to 1.32%. In general, Proteobacteria abundance decreased in both the AT and LT groups, whereas Firmicutes abundance increased to variable degrees. In general, over S0–S48, Bacteroidetes abundance increased in the LT group but decreased in the AT group, and Actinobacteria abundance decreased in the LT group but increased in the AT group. The Firmicutes-to-Bacteroidetes (F: B) ratio of the gut bacterial communities was approximately 7.09 at PH, and it increased to 18.24 at PT. At PR, the F: B ratio considerably increased to 127.88. During the simulated sales phase, the F: B ratio in the LT group decreased from 13.29 at S0 to 7.28 at S48, whereas that in the AT group increased from 30.44 at S0 to 115.22 at S48.

As depicted in Figure 4E, an analysis of the OTUs revealed a total of 13 core shared OTUs. The PH group (post-harvest) exhibited 462 unique OTUs, while the PT group, after transportation, displayed 500 unique OTUs. Following temporary respite (PR), the number of unique OTUs decreased to 177. During the subsequent 48 h simulated sales phase, the AT group showed an increase in unique OTUs from 55 to 77, whereas the LT group exhibited a rise from 121 to 175. LEfSe analysis (Figure 4F,G) identified 30 species significantly enriched in the AT group and 9 in the LT group, with 5 species exhibiting statistically significant differences (*p* < 0.05).

#### 3.3.3. Predictive Analysis of PICRUSt2 Function Pathways in Gut Microbiota

**Figure 5:**
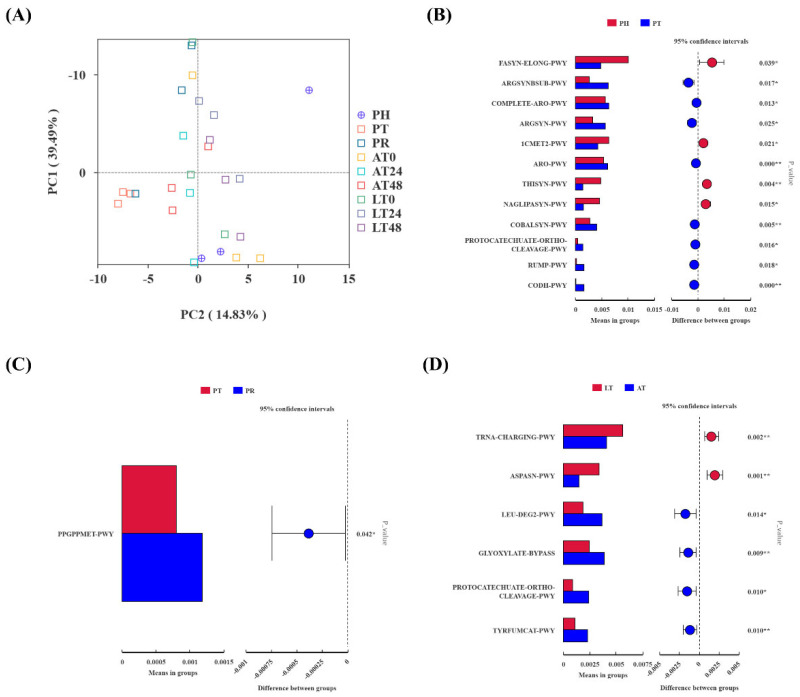
PCA plot (**A**) illustrating overall differences in predicted KEGG pathways among groups; *t*-test plots ((**B**): PT vs. PH; (**C**): PR vs. PT; (**D**): AT vs. LT) indicating significantly different pathways based on PICRUSt2 predictions of shrimp gut microbiota.. PH: post-harvest; PT: post-transport; PR: post-respite; LT0, LT24, and LT48: 0, 24, and 48 h, respectively, in AT group; AT0, AT24, and AT48: 0, 24, and 48 h, respectively, in AT group. ** indicates a significant correlation at the 0.01 level, * indicates significant correlation at the 0.05 level.

The predictive analysis of PICRUSt2 function in shrimp gut microbiota is shown in Figure 5. The PCA analysis (Figure 5A) demonstrated a distinct separation between the PT and PR groups and the other groups.

The *t*-test analysis of the KEGG pathway predictions from PICRUSt2 revealed significant alterations in pathways during the supply chain process. Figure 5B–D show the differential pathways notably affected. The transport phase influenced several physiological processes, including energy metabolism, amino acid synthesis, antioxidant stress, and cell repair, resulting in the upregulation of eight pathways and the downregulation of four pathways. The upregulated pathways include argsynbsub-pwy (L-arginine biosynthesis II via acetyl cycle), complete-aro-pwy (super pathway of aromatic amino acid biosynthesis), sargsyn-pwy (L-arginine biosynthesis I via L-ornithine), aro-pwy (chorismate biosynthesis I), cobalsyn-pwy (adenosylcobalamin salvage from cobinamide I), protocatechuate-ortho-cleavage-pwy (protocatechuate degradation II via ortho-cleavage pathway), rump-pwy (formaldehyde oxidation I), and codh-pwy (reductive acetyl coenzyme A pathway), the four downregulated pathways are fasyn-elong-pwy (fatty acid elongation-saturated), 1cmet2-pwy (N10-formyl-tetrahydrofolate biosynthesis), thisyn-pwy (super pathway of thiamine diphosphate biosynthesis I), and naglipasyn-pwy (lipid IVA biosynthesis) (Figure 5B). In the PR group, the ppGppmet-pwy (ppGpp biosynthesis) pathway was significantly upregulated (Figure 5C). The results of the *t*-test analysis between the AT and LT groups are shown in Figure 5D. The LT group exhibited upregulation of two pathways: tRNA-charging-pwy (tRNA charging) and aspasn-pwy (super pathway of L-aspartate and L-asparagine biosynthesis). The four pathways leu-deg2-pwy (L-leucine degradation I), glyoxylate-bypass (glyoxylate cycle), protocatechuate-ortho-cleavage-pwy (protocatechuate degradation II-ortho-cleavage pathway), and tyrfumcat-pwy (L-tyrosine degradation I) showed downregulation in the LT group.

#### 3.3.4. Correlation Analysis Between Differential Species, Differential Pathways, and Phenotypic Index in the LT Group

**Figure 6:**
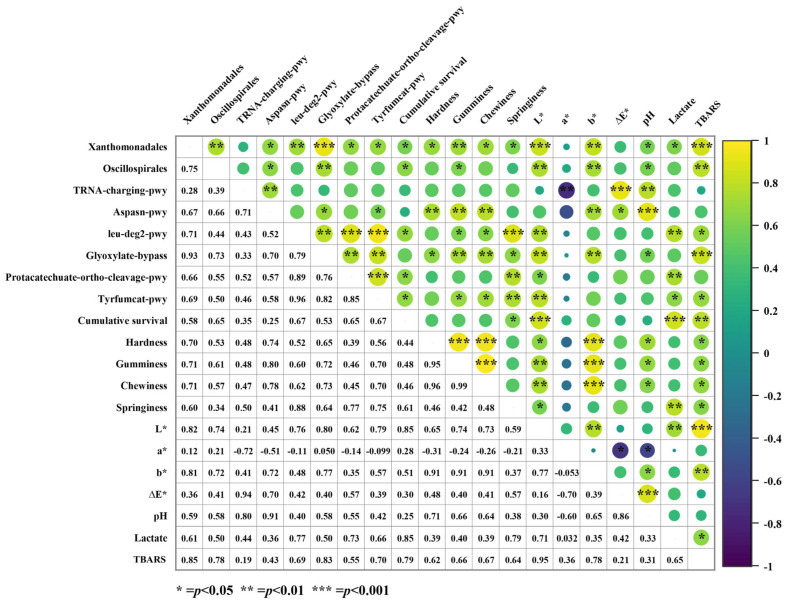
Correlation analysis between differential species in the LT group, differential pathways, and phenotypic indices. Numbers on lower left represent the corresponding correlation coefficients, while upper right indicates statistical significance. *** indicates significant correlation at the 0.001 level; ** indicates a significant correlation at the 0.01 level; * indicates significant correlation at the 0.05 level.

To further identify key biomarkers of low-temperature adaptation associated with shrimp phenotypic traits (survival and muscle quality), although LEfSe analysis identified five differentially abundant species, we focused on *Oscillospirales* and *Xanthomonadales* from the LT group for further analysis. The correlation analysis between differential species, the differential pathways, and the phenotypic indices are presented in Figure 6.

In the LT group, the abundance of *Xanthomonadales* was significantly and positively correlated with the aspasn-pwy, leu-deg2-pwy, glyoxylate-bypass, protocatechuate-ortho-cleavage-pwy, and tyrfumcat-pwy pathways. *Xanthomonadales* were also significantly and positively correlated with most phenotypic parameters, including cumulative survival rate, muscle texture, L*, b*, pH, and TBA values (*p* < 0.05). In contrast, *Oscillospirales* showed a significant positive correlation with the aspasn-pwy pathway, glyoxylate-bypass pathway, muscle hardness, springiness, L*, b*, pH, and TBA values (*p* < 0.05). Additionally, *Xanthomonadales* and *Oscillospirales* were also significantly positively correlated (correlation coefficient = 0.75, *p* < 0.01).

Regarding the KEGG-enriched differential pathways, leu-deg2-pwy showed a significant positive correlation with the glyoxylate-bypass, protocatechuate-ortho-cleavage-pwy, and tyrfumcat-pwy pathways. Three pathways (leu-deg2-pwy, glyoxylate-bypass, and tyrfumcat-pwy) were significantly positively correlated with cumulative survival rate, texture, L*, and TBARS values (*p* < 0.05). The aspasn-pwy and tRNA-charging-pwy pathways were significantly positively correlated, with the aspasn-pwy pathway showing a significant positive correlation with texture (hardness, gumminess, and chewiness), b*, and pH. In contrast, the tRNA-charging-pwy pathway was significantly negatively correlated with the a* value (correlation coefficient = −0.72, *p* < 0.01).

Regarding phenotypic indicators, survival was positively correlated with the L* value and TBA value (correlation coefficients of 0.85 and 0.79, respectively), while the L* value showed a significant positive correlation with texture (*p* < 0.05).

## 4. Discussion

The cumulative survival rate is a key indicator of transport efficiency, reflecting stress damage in shrimp. The high mortality rates observed during the transportation phase are likely attributable to rough handling, high stocking density, localized hypoxia, and physiological injuries induced by harvesting, all of which impose severe stress that may exceed the shrimp’s adaptive capacity. After the 24 h mark of simulated sales, the cumulative survival rate stabilized, likely due to the activation of adaptive strategies in shrimp, which gradually restored physiological homeostasis, leading to a reduction in mortality [34]. A study reported that in the water-preserved transport of *Macrobrachium rosenbergii*, survival rates were significantly higher at 21 °C compared with 26 °C [35]. Similarly, the LT group in this study exhibited the same effect. Muscle pH is positively associated with water-holding capacity and hardness and is influenced by metabolic byproducts such as lactate. As a key intermediate of glycolysis and mitochondrial respiration, lactate contributes to pH regulation and serves as an important substrate for gluconeogenesis [36]. Stress triggers glycolytic energy production to meet heightened energy demands, as seen in stressed Atlantic salmon [11]. At PT, stress activates the neuroendocrine system [37], AMP-activated protein kinase (AMPK) [9], and pyruvate kinase (PK) [38], promoting glycolysis and lactate accumulation due to increased ATP turnover and oxidative stress. During starvation, shrimp shift to alternative energy sources, generating lactate [39]. The decrease in lactate from 24 to 32 h suggests upregulated gluconeogenesis or lactate–phenylalanine complex formation, which suppresses appetite [40]. TBARSs represent an important indicator for evaluating lipid oxidation and flavor deterioration [41]. Their increase is primarily due to the oxidation of polyunsaturated fatty acids (PUFAs) in cell membranes, leading to the formation of toxic peroxides such as MDA [42]. Excess MDA induces apoptosis and activates immune responses [43], contributing to muscle degradation. Research shows that under stress, fish increase unsaturated fatty acids to improve cell membrane fluidity, but this also causes oxidative stress [44]. Furthermore, studies have shown that fatty acid oxidation under stress conditions serves as an important energy source for the body [45]. However, the oxidation of PUFAs (such as eicosapentaenoic acid and docosahexaenoic acid) can also promote protein oxidation in muscle, and the resulting damage to the protein structure likely affects the flavor and texture of the muscle [46]. Notably, TBARS levels remained low throughout the live supply chain and did not reach thresholds known to affect flavor, which is likely due to the inherently low lipid content of *P. vannamei*. Color and texture are important quality parameters for shrimp. Consumers prefer brighter, firmer, and more elastic muscles [47]. In our study, muscle brightness deteriorated at PT, which is consistent with findings related to crowding stress [10]. Stress-induced activation of the prophenoloxidase system reduces L* values [48]. Muscle texture deteriorated primarily due to pH drop [49], cortisol release [50], and protease activation [51]. During the sales phase, texture worsened in the AT group, but was less affected in the LT group, possibly due to inhibited protease and phenoloxidase (PO) activity at cold temperatures [52].

Predominant phyla in the intestine included Proteobacteria, Firmicutes, Actinobacteria, and Bacteroidetes, consistently with previous studies [53,54]. The high abundance of Gammaproteobacteria in adult shrimp intestines (up to 40%) is characteristic of gut dysbiosis [55,56]. Actinomycetes are crucial to intestinal homeostasis, and their rapid growth suggests potential gut dysregulation. This study also found that the dominance shifted from Proteobacteria (at PH) to Actinobacteria (at PT) and Firmicutes (at PR). In *Marsupenaeus japonicus* infected with WSSV, an increase in the abundance of Firmicutes over Proteobacteria was observed, suggesting that Firmicutes may play a key role in disease resistance [57]. However, under adverse stress conditions, the dominance of the gut microbiota in most aquatic animals shifts from Firmicutes to Proteobacteria, as seen in sea cucumbers under high-density, high-temperature, and low-salinity stress [33], as well as in *Rachycentron canadum* under 14 days of hypoxic stress [32]. These studies indicate that the evolution of dominant gut microbes is closely linked to different stress conditions, likely driving shifts in the host’s metabolic processes to cope with stress. Also, during the simulated sales phase, Proteobacteria and Firmicutes were the dominant phyla; however, during this phase, Proteobacteria abundance decreased, whereas Firmicutes abundance increased. Both of these dominant phyla are closely associated with the host’s carbohydrate and amino acid metabolism [58]. The bioactive metabolites they produce can communicate via the gut–muscle axis, influencing the nutrient metabolism and quality of muscle tissue [59]. A higher F: B ratio enhances intestinal fat metabolism and energy uptake in the host and increases fatty acid content in shrimp muscles [60]. Healthy animals have higher alpha diversity and a higher number of OTUs than diseased animals [61,62]. Enhanced gut health in the LT group, reflected by increased Bacteroidetes and OTUs and a higher Simpson index, suggests improved digestive efficiency and muscle quality [15]. Under starvation stress, suppressed digestive activity in shrimp triggers gut microbiota-mediated compensation and lipid metabolism [63], such as Firmicutes proliferation. Starvation is an inherent part of the live shrimp supply chain, making it difficult to separate its effects from post-harvest treatments. However, the lack of a starvation-only control remains a limitation. Furthermore, changes in water quality may affect shrimp stress responses, gut microbiota composition, and muscle quality. Future studies could integrate water quality and shrimp physiological data before and after transport to validate the impact of gut microbiota on muscle quality.

External conditions can change the structure of the intestinal flora, and these variations can drive the characterization of host metabolic function pathways [33]. The greater the number of unique OTUs retained, the greater the number of functions that help shrimp resist stress injury and metabolic adaptation. Transport increased the number of OTUs, likely by upregulating arginine and antioxidant pathways while downregulating fatty acid and vitamin synthesis. This indicates a shift in metabolic priorities to cope with transport-induced stress and resource limitations, such as oxygen deficiency. The ppGppmet-pwy pathway, which synthesizes guanosine 3′,5′-bisphosphate (ppGpp), regulates metabolic balance, mitigates stress damage, and enhances resilience by modulating energy homeostasis and the expression of antioxidant genes [64]. This suggests that the respite phase reshapes the gut microbiome’s function, mitigating transport-induced damage by enhancing the synthesis and metabolism of ppGpp. The leu-deg2-pwy and glyoxylate-bypass pathways are likely crucial to maintaining the balance of carbon and nitrogen metabolism [65]. The aspasn-pwy pathway is primarily involved in the synthesis of aspartic acid or arginine and can relieve stress [66]. In addition, arginine and aspartic acid can improve the hardness and elasticity of muscles [67]. *Xanthomonadales*, belonging to the Proteobacteria, and *Oscillospirales*, belonging to the Firmicutes, have been evaluated as potential probiotics in aquaculture [68]. Correlation analysis revealed a strong relationship between the differential species *Xanthomonadales* and *Oscillospirales* in the LT group and the phenotypic indicators of shrimp, particularly *Xanthomonadales*. In the LT group, *Oscillospirales* and *Xanthomonadales* likely maintain the high survival rate and quality of shrimp by reducing carbon and nitrogen metabolism and enhancing antioxidant stress resistance [69]. Although they are positively correlated with TBARS values, it is hypothesized that they may produce oxidation products (e.g., MDA) through metabolism itself, which may directly lead to elevated TBARS values, as TBARS values also show a positive correlation with some pathways (e.g., glyoxylate-bypass). In future studies, metabolomic approaches could help further investigate how these strains affect shrimp metabolism, shedding light on their functional roles and evaluating their potential as probiotics.

Finally, it is essential to integrate and discuss these indicators to evaluate the changes in the live supply chain and derive practical insights from these changes. Among the various stages of the supply chain, the transportation phase exhibited the greatest reduction in survival rate, during which the shrimp countered the associated stress by enhancing gut microbiota-mediated antioxidant defenses, stress-resistance mechanisms, and energy metabolism pathways. Meanwhile, we found that during the simulated marketing period, muscle quality changed as follows: survival stabilized and TBARSs increased after the 24 h mark, whereas L* decreased and a* increased after the 40 h mark. Therefore, the transport, 24 h sales, and 40 h sales stages were identified as three potential critical control points. These key points can be used to optimize conditions or adjust sales strategies in the live shrimp supply chain to maximize shrimp quality and shelf life. Moreover, post-transport respite effectively alleviated transport stress and gut microbiota dysfunction, indicating that a standardized respite procedure is beneficial for shrimp survival, especially during longer-distance transport. During the simulated sales period, the LT group exhibited higher survival rates and better quality parameters and downregulated the F: B ratio and carbon–nitrogen metabolism functions in the gut. These findings demonstrate that a low-temperature environment enhances shrimp stress resistance and stabilizes the interactions between the gut microbiota and the host. In addition to the group standard T/SZS 3016-2020, the fish transportation technical specification (T/HSKX 002-2021) [70] recommends that live fish transportation temperatures should not exceed 24 °C in summer. These support our finding that maintaining 23 °C in the LT group is an effective, feasible, and industry-aligned strategy for improving shrimp survival and quality during marketing. Notably, the enrichment of *Xanthomonadales* and *Oscillospirales* in the LT group, along with their positive correlation with survival rates and muscle quality indicators, suggests that these bacterial taxa could serve as biomarkers for assessing shrimp adaptation to low temperatures and the maintenance of muscle quality. This undoubtedly provides essential tools and new perspectives for future gut microbiota-based muscle quality control strategies.

## 5. Conclusions

The transport, 24 h sales, and 40 h sales stages were identified as three potential critical control points. Meanwhile, post-transport respite was effective in relieving transport stress and is recommended for standardization in the live supply chain. Notably, the LT group (23 °C) showed higher survival, resulted in higher muscle hardness and pH, and reduced gut-mediated metabolic levels, making it an effective and feasible solution. We speculate whether the improved physiological condition of the LT group might be related to the enrichment of *Xanthomonadales* and *Oscillospirales*, which could serve as biomarkers for muscle quality. As this study focused on short-distance transport, further validation over longer distances is needed. In the future, integrating water quality parameters, shrimp physiological indicators, and metabolomics will help clarify host–microbiota interactions, thereby advancing microbiota-based quality control strategies in the live supply chain and aquaculture.

## Data Availability

Raw data from the study are included in the text. For further inquiries, please contact the corresponding author.
